# Acetabular and sacral insufficiency fractures in a patient with a long-term history of Alendronate consumption; a case report

**DOI:** 10.1186/s12891-023-06327-8

**Published:** 2023-03-22

**Authors:** Kaveh Gharanizadeh, Hadi Ravanbod, Amir Aminian, Saeed Hatami, Ali Sherafati Chaleshtori, Soroush Kazerani

**Affiliations:** 1 Shafa Orthopedic Hospital, Iran University of Medical Science, Tehran, Iran; 2 Bone and Joint Reconstruction Research Center, Department of Orthopedics, School of Medicine, Iran University of Medical Sciences, Tehran, Iran; 3 Bone and Joint Reconstruction Research Center, Shafa Orthopedic Hospital, Iran University of Medical Sciences, Tehran, Iran; 4 Department of Orthopedic Surgery, School of Medicine, Isfahan University of Medical Science, Isfahan, Iran

**Keywords:** Bisphosphonate therapy, Alendronate, Sacral and pubic insufficiency fracture

## Abstract

**Background:**

Long-term Bisphosphonate consumption has been reported to be associated with the incidence of atypical or insufficiency fracture, particularly in the proximal femur. We observed a case of acetabular and sacral insufficiency fractures in a patient with a long-term history of Alendronate consumption.

**Case presentation:**

A 62-year-old woman was admitted with a complaint of pain in right lower limb following low-energy trauma. The patient had a history of Alendronate consumption for more than 10 years. The bone scan revealed increased radiotracer uptake in the right side of the pelvic, proximal right femur, and sacroiliac joint. The radiographs showed type 1 sacrum fracture, acetabulum fracture with femur head protrusion into the pelvis, quadrilateral surface fracture, fracture of the right anterior column, and right superior and inferior pubic fracture. The patient was treated with total hip arthroplasty.

**Conclusion:**

This case highlights the concerns regarding long-term bisphosphonate therapy and its potential complications.

## Background

Bisphosphonates are a class of drugs commonly used to treat senile and postmenopausal osteoporosis due to their ability to reduce the risk of bone fractures elsewhere in the body besides the spine. Its mechanism is based on the anti-resorptive activity of bone, which reduces osteoclast numbers, reduces osteoclast function, increases apoptosis, and prevents bone destruction. Strong and persistent inhibition of resorption disrupts normal bone remodeling, which is required to maintain bone quality, resulting in excessive mineralization and increased bone density. This process makes bones more rigid, leading to the accumulation of micro cracks, increased micro fractures, and possibly the development of fractures, especially when combined with extrinsic factors like the action of asymmetric mechanical burden in the femur [1, 2]. Other drugs, such as proton-pump inhibitors, glucocorticoids, and/or estrogens, may also be correlated with the suppression of bone remodeling. Patients who have been taking bisphosphonates for 6 years or more have been found to have an increased risk of atypical fractures due to non-osteoporotic femur insufficiency, according to multiple reports in the medical literature [3]. In this study, we report our observation regarding a case of acetabulum and sacrum insufficiency fracture in a patient with a long-term history of Alendronate medication.

## Case presentation

**Fig. 1:**
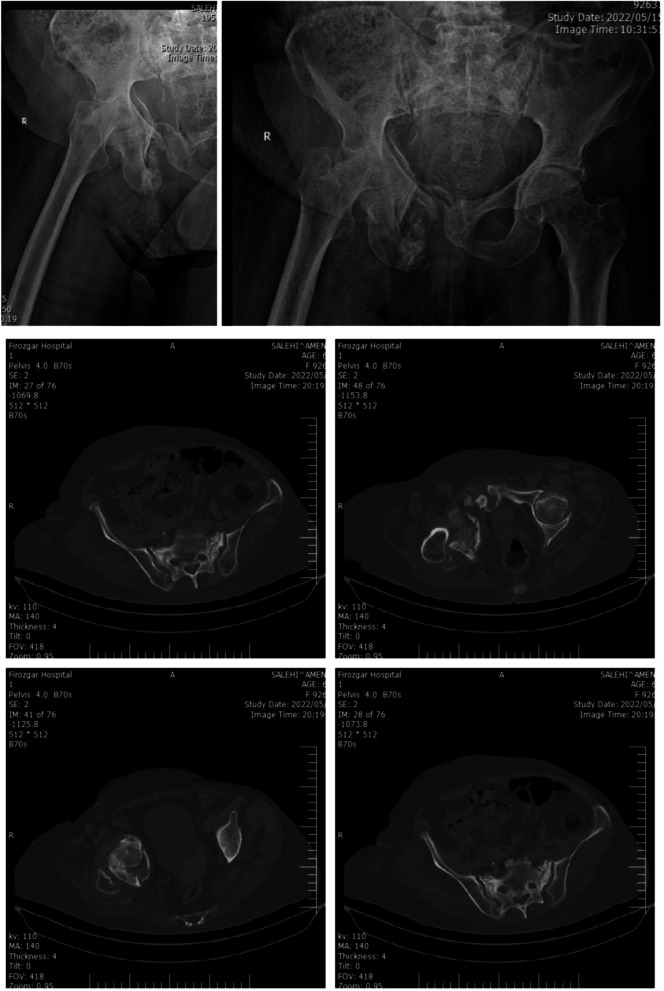
Patient’s pre operation radiographs

**Fig. 2:**
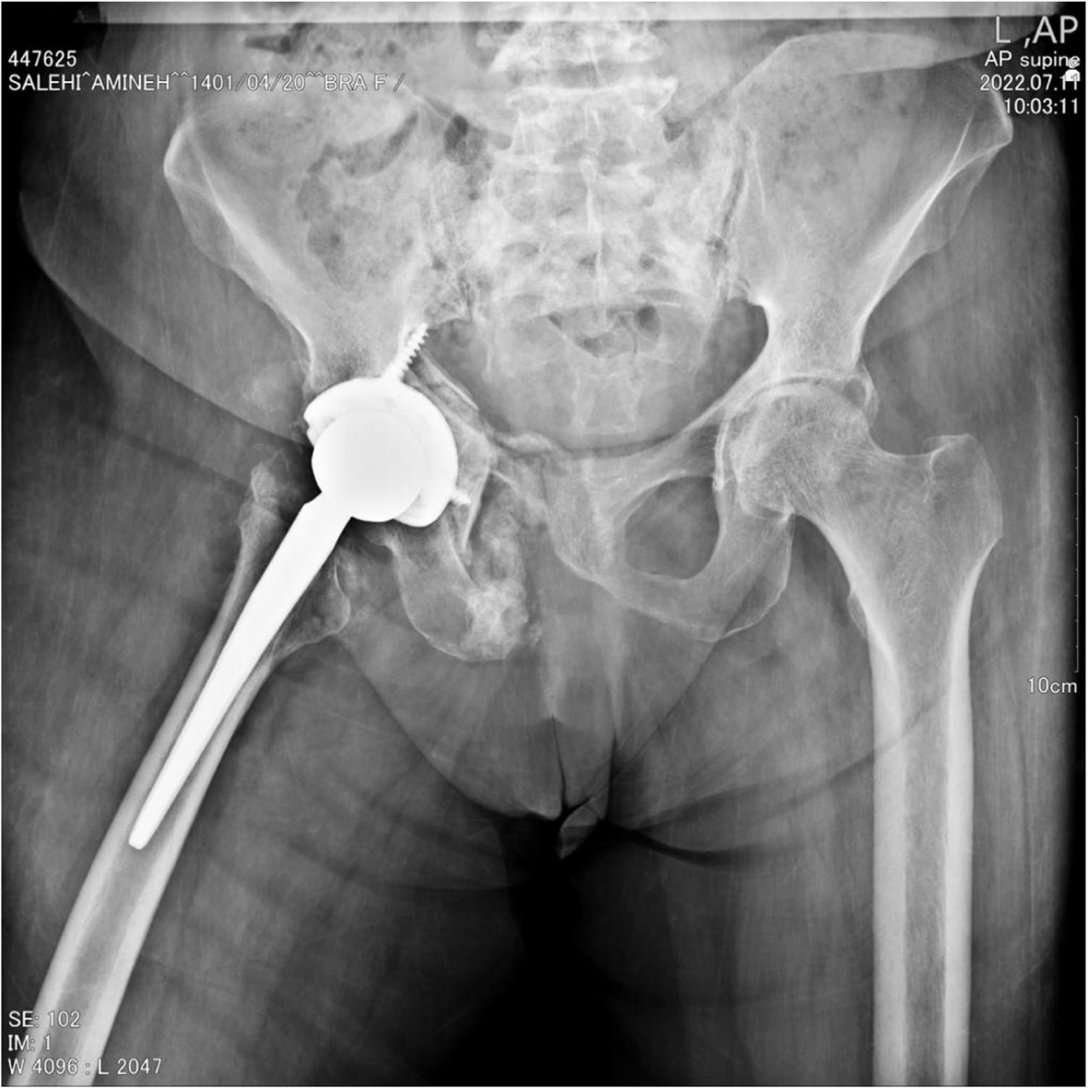
Patient’s post operation radiographs

A 62-year-old woman was admitted with a complaint of pain in her right lower limb following low-energy trauma (sitting position from a standing position). The pain has gradually intensified over the last 2 months so she was unable to walk. She had a history of hypertension, rheumatoid arthritis, and Parkinson’s disease. In history taking, she mentioned using Alendronate medication (70 mg per week) for almost 10 years. Also, she was receiving Methotrexate 100 mg every week, Prednisolone 2.5 mg daily, Calcium and Vitamin D daily, Teriparatide vial 8 units daily, Amlodipine 5 mg daily, Carvedilol 6.25 daily, Levodopa and Benserazide hydrochloride, and Folic acid daily. Based on the visual analogue scale (VAS), the patient stated her pain was 8 out of 10. Due to the severe pain, any medical or diagnostic tests were impossible. The patient’s hip range of motion (ROM) was extremely limited. According to bone mineral densitometry, T-scores for the femoral neck, total hip, and lumbar spine were − 0.9, − 1, and − 2.4, respectively. The patient wasn’t osteoporotic. The patient’s bone scan reveals increased radiotracer uptake in the right side of the pelvis (superior and inferior rami of pubis and right acetabulum), proximal right femur, and sacroiliac joint (sacrum). The radiographs showed type 1 sacrum fracture, acetabulum fracture with femur head protrusion into the pelvis, quadrilateral surface fracture, fracture of the right anterior column, and right superior and inferior pubic fracture (Fig. 1). Due to the suspicion of malignancy and rule out multiple myeloma, we took a biopsy of the pubis and it showed no evidence of a tumor or multiple myeloma. The patient was treated with total hip arthroplasty (THA) (Fig. 2).

## Discussion and conclusion

In the past decades, nearly all pelvic fractures were related to high-energy traumas in young, otherwise healthy people. This pattern is shifting in recent decades so that these injuries are more commonly seen in the elderly and are linked to low-energy impacts as well as spontaneous fractures in people with severe osteoporosis. As of now, bisphosphonates are the gold standard for treating osteoporosis [2, 4–6]. Despite their widespread application in medicine, many questions remain unanswered, particularly with regard to the most effective methods of care. For instance, what is the best therapy duration? A recent comprehensive study reveals that after 3 years of treatment, bisphosphonates show positive impacts on bone mineral density and fracture prevention. Importantly, beneficial treatment effects were maintained and observed after the discontinuation of bisphosphonates administered for almost 3 years. There was no difference in fracture risk between postmenopausal women with osteoporosis who continued medication after 5 years and those who discontinued treatment [7–10].

The potential side effects of long-term bisphosphonate consumption, mainly atypical femoral fracture, have also been the subject of many discussions over recent years. However, there is a very limited amount of research available on the association between bisphosphonates consumption and pelvic insufficiency fractures. The nature of any evidence is anecdotal. No conclusive evidence of this connection was found in a recent meta-analysis of Phase III trials. However, most of the examined randomized controlled studies were conducted prior to the clinical discussion of these atypical fractures. It is likely that studies are underpowered to detect an association between atypical fractures and bisphosphonates since the frequency of atypical fractures is low compared to the number of patients taking the drug [11, 12].

To date, the majority of publications on atypical fractures resulting from long-term bisphosphonate treatment have documented fractures of the subtrochanter or diaphysis of the femur [13]. To the best of our knowledge, there is one article describing a case of sacral and pubic insufficiency fracture in a 67-year-old woman who had 4 years history of Bisphosphonate therapy, one study describing a pelvic fracture in a 63-year-old woman after long-term Alendronate therapy, and a case of acetabular fracture in a 77-year old woman after 6 years of Bisphosphonate therapy [1–3]. In this study, we reported a case of acetabular and sacral insufficiency fractures in a 62-year-old female with a 10-year history of Alendronate consumption. It is of note that we only reported our observation and the causality relationship between insufficiency fractures and Alendronate use cannot be concluded by this report.

In summary, this article reports a rare case of the acetabulum and sacral insufficiency fracture and highlights the concerns regarding long-term bisphosphonate therapy. Similarly, it highlights the need for analytical investigations with a larger sample group to confirm the causality between fractures and Alendronate use.

## Data Availability

Data sharing is not applicable to this article as no datasets were generated or analysed during the current study.

## Abbreviations

VAS: Visual analouge scale
ROM: Range of motion
THA: Total hip arthroplsaty
